# An Adaptive Deep Ensemble Learning for Specific Emitter Identification

**DOI:** 10.3390/s25196245

**Published:** 2025-10-09

**Authors:** Peng Shang, Lishu Guo, Decai Zou, Xue Wang, Pengfei Liu, Shuaihe Gao

**Affiliations:** 1 National Time Service Center, Chinese Academy of Sciences, Xi’an 710600, China; shangpeng@ntsc.ac.cn (P.S.); zoudecai@ntsc.ac.cn (D.Z.); liupengfei@ntsc.ac.cn (P.L.); gaoshuaihe@ntsc.ac.cn (S.G.); 2 Key Laboratory of Time Reference and Applications, Chinese Academy of Sciences, Xi’an 710600, China; 3 School of Astronomy and Space Science, University of Chinese Academy of Sciences, Beijing 100049, China; 4 Institute of Information Sensing, Xidian University, Xi’an 710600, China; xuewang@xidian.edu.cn

**Keywords:** specific emitter identification, radio frequency fingerprints, adaptive deep ensemble learners, hybrid losses

## Abstract

Specific emitter identification (SEI), which classifies radio transmitters by extracting hardware-intrinsic radio frequency fingerprints (RFFs), faces critical challenges in noise robustness, generalization under limited training data and class imbalance. To address these limitations, we propose adaptive deep ensemble learning (ADEL)—a framework that integrates heterogeneous neural networks including convolutional neural networks (CNN), multilayer perception (MLP) and transformer for hierarchical feature extraction. Crucially, ADEL also adopts adaptive weighted predictions of the three base classifiers based on reconstruction errors and hybrid losses for robust classification. The methodology employs (1) three heterogeneous neural networks for robust feature extraction; (2) the hybrid losses refine feature space structure and preserve feature integrity for better feature generalization; and (3) collaborative decision-making via adaptive weighted reconstruction errors of the base learners for precise inference. Extensive experiments are performed to validate the effectiveness of ADEL. The results indicate that the proposed method significantly outperforms other competing methods. ADEL establishes a new SEI paradigm through robust feature extraction and adaptive decision integrity, enabling potential deployment in space target identification and situational awareness under limited training samples and imbalanced classes conditions.

## 1. Introduction

SEI has aroused great interest among researchers in recent years. Its main purpose is to classify the radio transmitters by extracting the RFFs, embedded in signals for the hardware imperfections. These imperfections include power amplifier (PA) nonlinear distortions, I/Q modulation errors and filter distortions [1]. SEI has extensive application in military and civil domains, such as spectrum management [2], self-organized networking [3], cognitive radio [4] and Internet of Things (IOT) device authentication [5,6].

Research on SEI via RFFs can be broadly categorized into the following two categories: hardware intrinsic imperfections-based and feature-based approaches. Hardware intrinsic imperfections-based classification [7,8,9] requires the accurate prior information about channel parameters and precise modelling of the hardware components, which is hard to be satisfied in non-cooperative scenarios. For real-world deployment, feature-based pipelines outperform alternatives through the following two distinct pathways: traditional algorithmic feature engineering or deep networks that autonomously derive task-optimized representations.

Traditional hand-crafted feature extraction algorithms mainly include power spectrum density [10,11], bi-spectrum analysis [12,13,14], time–frequency transformations, such as short-time Fourier transform (STFT) [15,16], Wigner–Ville distribution (WVD) [17,18], Hilbert–Huang transform (HHT) [19,20,21] and wavelet transform [22,23]. There is still other research about nonlinear features [24,25,26] and image features that convert the time domain I/Q samples to constellation maps [27] or differential constellation maps [28,29]. Time–frequency transformations convert raw signals into image-formatted representations on a 2D plane. However, such high-dimensional representations often necessitate complex classifiers while relying on substantial training samples [30,31]. Artificially engineered RFFs are proved to be labor-intensive and time-consuming due to their dependence on hand-crafted feature extraction algorithms requiring expert experience and knowledge of specific communication protocols [32,33].

Recently, with the development of deep learning, deep learning networks have achieved remarkable performance in SEI. These networks include CNN [15,34], residual network (ResNet) [35,36] and recurrent neural network (RNN) [37,38]. Newer networks like complex-value convolutional neural network (CVCNN) [39,40], attention mechanisms [41,42,43] and transformer [44] are applied to RFFs to try to extract features better. However, most of these networks are validated under the adequate training samples and balanced class distributions. Besides, the generalization of a sole network can be vulnerable to noise and class imbalance compared to the integration of heterogeneous networks.

Deep ensemble learning-based approaches are a good way to address the above-mentioned issues. In [45], a composite ensemble learning (CEL) integrates wavelet-based denoising with hybrid feature extraction, combining manually engineered features (from first/second derivatives of denoised signals) and automatically learned representations (via a cascaded CNN–LSTM (long short-term memory) network) to enhance feature diversity and robustness. In [46], an ensemble learning method with convolutional neural networks (CNNEL) processes three parallel representations—raw I/Q signals, STFT and wavelet transforms—through separate convolutional neural networks, with final identification achieved via averaging or voting across model outputs. In [47], two deep ensemble algorithms, hierarchical and multi-stage feature fusion (HMSFF) and cooperative decision (Co_decision) extract complementary RFFs including differential constellation trajectory amplitude, energy spectrum and spectral symmetry, enabling multi-stage feature fusion or cooperative inference. In [48], a deep ensemble learning method for SEI is proposed by feeding amplitude, phase and spectral asymmetry features into parallel CNNs, aggregating predictions through output averaging.

Motivated by the above research, an adaptive deep ensemble learning (ADEL) framework is proposed. Its main ideal is to leverage the merits of the following three deep networks: CNN for local spatiotemporal feature extraction, MLP for global feature interactions and transformers for long-range contextual dependency modeling to foster feature robustness. Besides, hybrid loss optimization strategies include reconstruction loss (preserving signal integrity), cross-entropy (sharpening decision boundaries) and center loss (compressing intra-class variance) to refine feature space geometry are adopted to get better feature generalization. Lastly, adaptive weighted inference is made by averaging the outputs of the three base learners weighted by the reconstruction errors of the testing signals. The effectiveness of the ADEL is also validated by extensive experiments and its accuracy recognition significantly outperforms other competing methods.

The contributions of this paper are as follows:

(1) Unified multi-representation fusion architecture: the ADEL framework integrates complementary feature representations across convolutional, perceptron and attention-based architectures, establishing a hierarchical fusion paradigm that fundamentally advances robust feature extraction for SEI;
(2) Geometrically constrained multi-objective optimization: hybrid loss coordination refines feature space geometry through simultaneous signal integrity preservation (reconstruction loss), discriminative boundary sharpening (cross-entropy) and intra-class variance compression (center loss), enabling theoretically grounded representation learning;
(3) Test-time adaptive inference mechanism: reconstruction-error-weighted fusion dynamically recalibrates ensemble decisions during inference phase, boosting the reliability of ensemble aggregation.

## 2. Dataset and Methods

### 2.1. Dataset

The benchmark dataset employs Automatic Dependent Surveillance–Broadcast (ADS-B) signals sourced from [39]. Operating at 1090 MHz, this open-access passive reception system [49] utilizes a Signal Hound SM200B (Signal Hound, Inc., Battle Ground, WA, USA) receiver paired with a 1090-MHz omnidirectional antenna for raw signal acquisition. High-performance computing demodulates these signals to construct the dataset, which includes aircraft identification codes (ICAO 24-bit addresses), spatiotemporal parameters (position, altitude, velocity) and auxiliary telemetry metadata. Crucially, sample categories are programmatically derived through aircraft ID decoding of demodulated data blocks. Preprocessed signals are formatted as I/Q samples at 50 MS/s sampling rate with each sample spanning 6000 points. The dataset encompasses 20 distinct aircraft classes, with full experimental dataset details available in [49].

**Figure 1:**
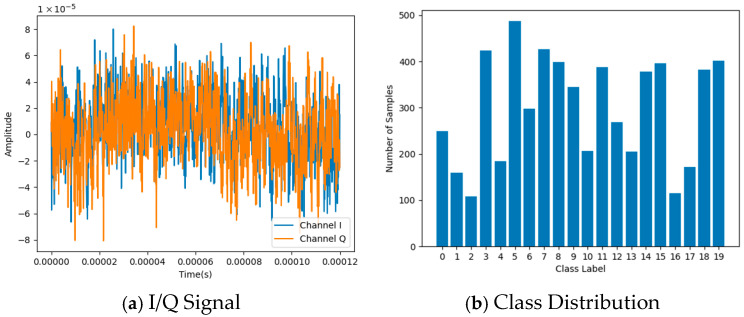
The data characteristics of the training set.

The experimental dataset comprises 5992 training samples and 2000 testing samples. Figure 1 illustrates both the I/Q signal characteristics and the class distribution of the training set. The ADEL framework directly ingests raw I/Q signals, eliminating preprocessing overhead while preserving signal integrity. Significant class imbalance exists, with Class 5 containing the maximum samples (*n* = 487) and Class 2 the minimum (*n* = 108), resulting in an imbalance ratio exceeding 4.5:1.

### 2.2. Methods

**Figure 2:**
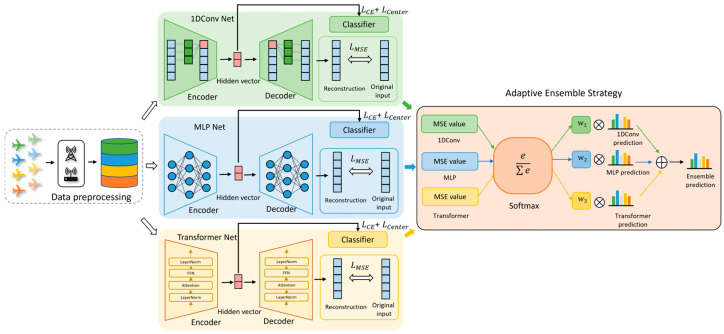
The overall framework of ADEL.

To address feature degradation under data scarcity and class imbalance as demonstrated in Figure 1, we propose the adaptive deep ensemble learning (ADEL) framework. ADEL integrates three architecturally heterogeneous base learners—CNN, MLP and transformer for robust feature extraction—each equipped with a dedicated decoder module. The core innovation resides in reconstruction-error-guided dynamic weighting, where decoder fidelity serves as a proxy for evaluating feature extraction competence, and hybrid loss optimization combining signal reconstruction integrity, decision boundary sharpening and intra-class variance compression. Crucially, parameter sharing of the classifier and decoder across learners enforces feature space alignment, enhancing discriminative capacity. The integrated framework is depicted in Figure 2.

Data preprocessing adopted here is to normalize the energy of each sample. Additionally, we also randomly split the dataset into training set, validation set and testing set with the percentage of 64%, 16% and 20%. The three base encoder learners and the shared classifier and decoder will also be given in Section 2.2.1. *L_CE_*, *L_Center_*, *L_MSE_* stands for categorical cross-entropy loss, the center loss and mean square error (MSE), respectively; their expressions will be given in the Section 2.2.2. The adaptive ensemble strategy to aggregate the predictions of the three base learners will be given in Section 2.2.3.

#### 2.2.1. Network Structures

The overall networks include the following three base learners: CNN, MLP and transformer for feature extraction. The decoder is to reconstruct the latent features learner by the base learners into original signal. The classifier is to make the final predictions according to the extracted latent features. The structure of the networks is implemented in Pytorch 1.7.0.

The CNN base learner (Table 1) employs a four-stage convolutional cascade. Each stage executes convolution, batch normalization, ReLU activation and 2 × 2 MaxPooling in sequence. Convolutional layers maintain uniform input/output channel dimensions except the first layer, which processes 2-channel I/Q inputs [batch_size, 2, 6000]. This design achieves progressive spatial abstraction while preserving discriminative features via subsampling.

The MLP base learner (Table 2) integrates a convolutional feature compressor performing spatial abstraction, a ReLU-activated FC layers for nonlinear feature transformation and a linear projection head generating final embeddings. This architecture facilitates efficient conversion from spatial representations to discriminative feature vectors while preserving signal fidelity.

The transformer base learner (Table 3) builds upon the CNN’s four-stage convolutional hierarchy, augmenting it with an additional transformer layer. The architecture incorporates four identical Conv-MaxPooling units (Conv1→MaxPooling1 cascades repeated ×4), though Table 3 displays only a single unit for conciseness. This hybrid design leverages CNN’s localized feature extraction while integrating transformer’s global dependency modeling capabilities.

Within each base learner, the feature extractor standardizes sample representations into a unified (48, 375) dimensional tensor, enabling both shared classifier deployment for consistent prediction generation and decoder compatibility for uniform signal reconstruction. The classifier module projects these encoded features onto the 20-dimensional class space, with outputs driving cross-entropy loss computation during training. For reconstruction, the decoder first expands feature dimensions to (48, 750) via a fully connected layer, establishing compatible scaling for subsequent operations. Three transposed convolutional layers with progressive up-sampling then restore the original signal characteristics—recovering both the 2-channel configuration and 6000-point temporal resolution—while maintaining end-to-end reconstruction fidelity. The details of the shared classifier and decoder are given in Table 4 and Table 5. The symbol ✗ indicates the hyperparameter is not required.

#### 2.2.2. Hybrid Loss Optimization

The optimization employs a hybrid loss function integrating the following three components: categorical cross-entropy loss, computed from weighted ensemble logits; MSE: averaged across three base learners; and center loss: similarly averaged to compress intra-class variance.

For an ensemble with learners ${\left\{f_{i}\right\}}_{i=1}^{3}$, the hybrid loss is:

(1) $$L_{total}=\frac{1}{3}\sum_{i=1}^{3}w_{i}L_{CE}\left(f_{i}\left(x\right),y\right)+\lambda \frac{1}{3}\sum_{i=1}^{3}{\left\|x-g_{i}\left(h_{i}\right)\right\|}^{2}+\lambda_{center}\frac{1}{3}\sum_{i=1}^{3}{\left\|h_{i}-c_{y}\right\|}^{2}$$

where x is the original signal, y is the ground truth label, $f_{i}\left(x\right)$ is the logits of the classifier, $h_{i}$ is the latent feature learned by the base learner, reconstruction errors $\epsilon_{i}={\left\|x-g_{i}\left(h_{i}\right)\right\|}^{2}$ determines weights $w_{i}=e^{-\epsilon_{i}}/\sum e^{-\epsilon_{i}}$, favoring learners with higher reconstruction fidelity. $c_{y}$ is the learnable centroid of each class, with learning rate *η*. Hyperparameters λ and $\lambda_{center}$ are to balance loss components during gradient-based optimization. $L_{CE}$ is the categorical cross-entropy and can be defined as follows:

(2) $$L_{CE}=-\sum_{i=1}^{C}y_{i}log\left(p_{i}\right)$$

where C is the number of classes, $y_{i}$ denotes the one-hot encoded ground truth label and $p_{i}$ represents the integrated model’s predicted probability for class i.

Model weights *θ* are updated via stochastic gradient descent with learning rate *η*:

(3) $$\theta_{t+1}=\theta_{t}-\eta \nabla_{\theta }L_{total}$$

#### 2.2.3. Adaptive Ensemble Strategy

Once the adaptive ensemble models are well trained, within the ensemble framework, the outputs of three base learners are weighted based on reconstruction errors from their decoder modules. Specifically, for a given sample, a base learner with smaller reconstruction error indicates more effective feature extraction, thus receiving higher weight. This work employs the Softmax function to compute weights for each base learner, transforming the negative reconstruction errors into a probability distribution that sums to unity across all learners.

Firstly, the reconstruction loss can be calculated. For each base learner i, compute the MSE between input signals and reconstructed outputs:

(4) $${MSE}_{i}=\frac{1}{n}\sum_{j=1}^{n}{\left(x_{i,j}-{\hat{x}}_{i,j}\right)}^{2}$$

where ${MSE}_{i}$ denotes the reconstruction error for the *i*-th base learner, $x_{i,j}$ represents the *j*-th original sample under test and ${\hat{x}}_{i,j}$ is its reconstructed counterpart.

Secondly, Softmax-based weight assignment transforms reconstruction errors into normalized weights using the formulation:

(5) $$w_{i}=\frac{e^{{-MSE}_{i}}}{\sum_{i=1}^{M}e^{{-MSE}_{i}}}$$

where *M* = 3 is the number of base learners. This formulation assigns higher weights to learners with smaller reconstruction errors through the negative exponential transformation.

Lastly, the ensemble prediction integration mechanism aggregates weighted outputs from all base learners through summation:

(6) $$F\left(x\right)=\sum_{i=1}^{M}w_{i}p_{i}\left(x\right)$$

where $p_{i}\left(x\right)$ is the prediction from the *i*-th base learner.

This weighting strategy dynamically leverages the strengths of each learner, prioritizing those with superior feature representation capabilities to enhance overall classification accuracy and robustness under varying signal conditions.

## 3. Results

To rigorously validate the efficacy of the proposed adaptive deep ensemble learning (ADEL) framework, comprehensive experiments are conducted against the following two categories of benchmark methods: state-of-the-art deep learning architectures for signal identification and advanced ensemble learning methodologies. This comparative analysis quantitatively demonstrates ADEL’s superiority under data scarcity and class imbalance conditions.

### 3.1. Experiment Setup and Evaluation Criteria

Deep learning architectures for RF signal processing demonstrate specialized innovations as follows: MCNet [50] leverages asymmetric convolutions with skip-connections for multi-scale spatiotemporal feature extraction. PETCGDNN [51] integrates phase-aware correction within lightweight CNN–GRU hybrids for parameter-efficient modulation recognition. Adapted 1D CNN/VGG [52] enables temporal signal processing, while MSCANet [53] orchestrates multi-scale attention for noise robustness. CVCNN [40] preserves IQ signal coupling via complex-valued operations, maintaining quadrature relationships lost in real-valued networks. FFTMHA [54] reconstructs high-frequency components through Fourier-attention mechanisms, enabling joint spectral–temporal modelling via RNN feature extraction.

The deep ensemble learning methodologies include a composite ensemble learning (CEL) in [45], an ensemble learning method with convolutional neural networks (CNNEL) in [46], two deep ensemble algorithms, hierarchical and multi-stage feature fusion (HMSFF) and cooperative decision (Co_decision) in [47], the deep ensemble learning method for SEI by multi-feature fusion in [48], feeding amplitude, phase and spectral asymmetry features into parallel CNNs, aggregating predictions through output averaging, and the lightweight transformer-based network GLFormer in [32].

To ensure a rigorous and fair comparison, all deep learning models in this study were trained under identical conditions using the Adam optimizer [55] with a fixed learning rate of 1.6 × 10^−4^. The training protocol consisted of 200 maximum epochs with an early stopping mechanism (patience = 10) to prevent overfitting, and a consistent batch size of 32 was maintained throughout. For hyperparameter optimization, we conducted a systematic grid search using Optuna, exploring the following parameter spaces: convolutional layer channels ranging from 32 to 128 in increments of 16, transformer attention heads selected from {4, 8, 16} and convolutional layers varying between 3 and 8. This standardized experimental design guarantees that any observed performance differences can be confidently attributed to architectural variations rather than training inconsistencies or parameter selection biases.

The evaluation metrics in the experiments are accuracy, precision, recall and macro-F1. The macro-F1 assigns equal weight to each category, making it particularly suitable for scenarios with class imbalance.

(7) $$accuracy=\frac{TP+TN}{TP+TN+FP+FN}$$

(8) $$precision=\frac{TP}{TP+FP}$$

(9) $$recall=\frac{TP}{TP+TN}$$

(10) $$macro-F1=\frac{1}{C}\sum_{i=1}^{C}F1_{i}$$

(11) $$F1_{i}=\frac{2\times Precision_{i}\times Recall_{i}}{Precision_{i}+Recall_{i}}$$

where *TP* denotes the true positive, *TN* represents true negative, *FP* stands for false positive, *FN* means false negative, *C* is the total number of classes and F1*_i_* denotes the F1-score corresponding to the *i*-th class. Following Equations (8)–(10), precision, recall and macro-F1 are computed for each class. The overall dataset metrics represent the weighted average of these per-class values.

### 3.2. Benchmarking Results

The benchmarking results are based on the following two benchmark methodologies: deep learning models and deep ensemble learning methods; their outcomes in terms of the evaluation metrics are given in Table 6. All experimental results are reported as the mean value ± standard deviation from five independent replicates. Our comparative analysis demonstrates ADEL’s statistically significant superiority over CNN/VGG. The 95% confidence intervals show complete separation between architectures (ADEL: 98.25 ± 0.86 vs. CNN/VGG: 96.58 ± 1.11), with no interval overlap indicating robust performance differences. The paired *t*-test yields highly significant results (t = 8.944, *p* = 0.00086). The extremely low *p*-value (*p* < 0.001) provides overwhelming evidence rejecting the null hypothesis, establishing that ADEL’s accuracy improvement is both statistically and practically significant. The results in Table 6 demonstrate the superiority of the ADEL framework. To facilitate comparison, the best performance achieved across all replicates is presented in the following sections.

ADEL demonstrates dominant performance across all evaluation metrics, achieving near-perfect scores that reflect exceptional classification consistency as follows: 0.9975 accuracy, 0.9975 precision, 0.9975 recall and 0.9975 macro-F1 score; this apparent equality stems from rounding at the 10^−4^ precision level. This equilibrium—maximizing true positive identification while minimizing false positives/negatives—exceeds existing methods by substantial margins. Specifically, ADEL outperforms the strongest baseline (CNN/VGG) by 2.55% in accuracy (0.9975 vs. 0.9720) and 2.52% in macro-F1 score (0.9975 vs. 0.9723), while surpassing weaker methods like FFTMHA [54] by 24.55% in accuracy.

Performance degradation in comparative methods (e.g., FFTMHA’s 0.7520 accuracy) primarily stems from training data scarcity and class distribution imbalance, which collectively exacerbate model overfitting. Notably, ensemble methods like CNNEL and multi-feature achieve comparable performance (e.g., multi-feature: 0.9575 accuracy; CNNEL: 0.9550 accuracy) through handcrafted multi-feature extraction and deep ensemble architectures. Nevertheless, ADEL establishes holistic superiority with +4% accuracy and +4.03% macro-F1 score over multi-feature, and +4.25% accuracy/+4.57% macro-F1 score over CNNEL. This advancement derives from ADEL’s heterogeneous deep network architecture, which enables end-to-end multi-scale representation learning, hybrid loss optimization and adaptive ensemble inference—eliminating manual feature engineering while enhancing generalization.

**Figure 3:**
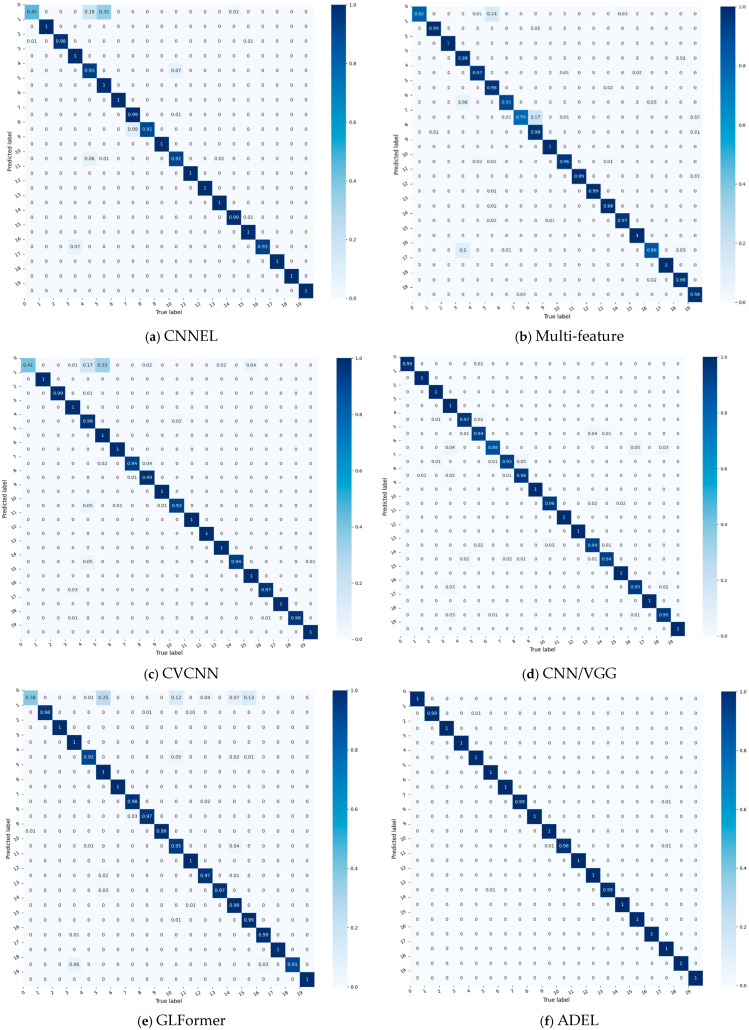
The comparative results of confusion matrices on ADS–B dataset.

For granular analysis of per-class accuracy, Figure 3 compares confusion matrices of four top-performing baselines (each exceeding 90% overall accuracy).

The confusion matrices of five models—four baselines (Figure 3a–d) and the proposed ADEL (Figure 3f)—reveal critical performance patterns through color-encoded probability distributions. CNNEL reveals critical model inequity with catastrophic failure in Class 0 (accuracy = 45%) alongside perfect performance in 12 classes. Multi-feature shows improved diagonal consistency (79–100%) yet suffers hazardous cross-class confusions (Class 5→0: 14%, Class 8→7: 17%, Class 4→16: 10%). CVCNN exhibits significant performance volatility across the 20 classes, with accuracy ranging from 41% to 100%. This 0.59 span represents severe inconsistency, particularly evidenced by the dramatic contrast between Class 0’s catastrophic failure (0.41 accuracy) and the perfect classification achieved for 10 categories (Classes 1, 3, 5, 6, 9, 11, 12, 15, 17 and 19). CNN/VGG model significantly outperforms all other baselines, demonstrating commendable stability: its minimum per-class accuracy reaches 88%, while 19 of 20 classes (>95%) exceed 93% accuracy. Crucially, 75% of classes (15/20) achieve > 95%, confirming robust generalization across categories. The confusion matrix of the proposed ADEL method demonstrates exceptional classification accuracy across all 20 categories, establishing its superiority over comparative methods. The model shows near-perfect accuracy: 100% for 16 classes and ≥98% for others (lowest: Class 10 at 98%).

**Figure 4:**
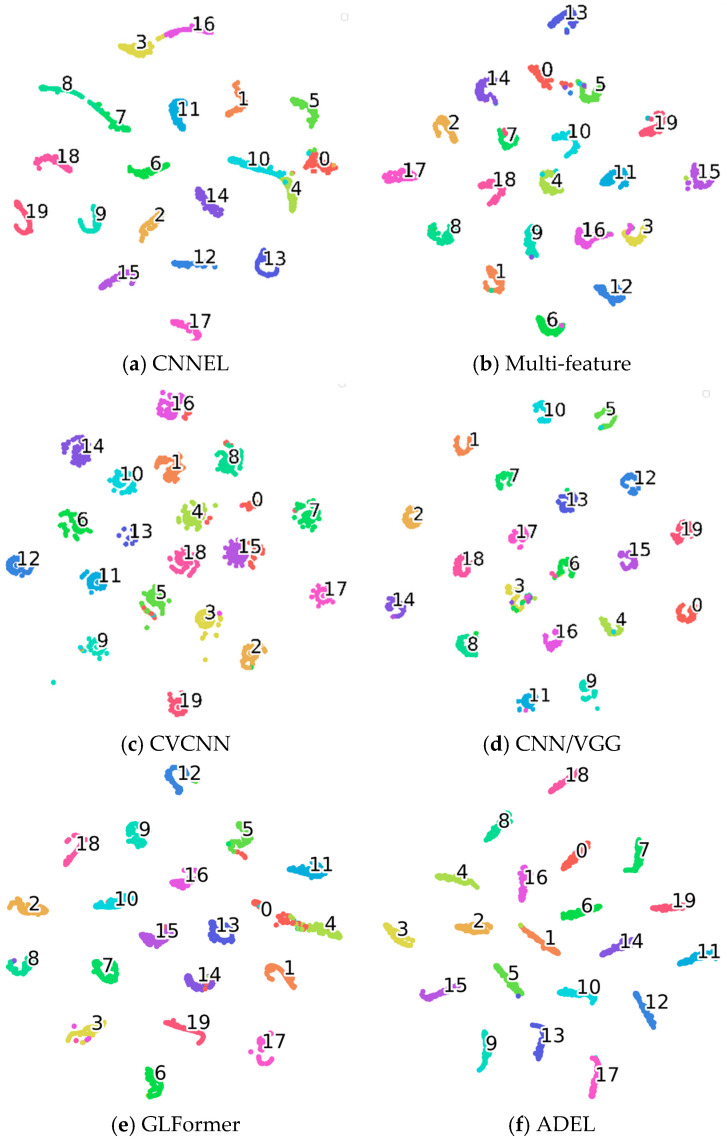
The t-SNE results of learned features on ADS–B dataset.

To gain deeper insight into the learned feature representations of each network, we perform dimensionality reduction on the 20-dimensional features from the final fully connected layer using t-Distributed Stochastic Neighbor Embedding (t-SNE). The quality of these feature representations is quantitatively evaluated via silhouette coefficients, with higher values indicating stronger discriminative power. Comparative deep features and silhouette scores (SC) for all networks are visualized in Figure 4 and given in Table 7, respectively, the number in the clusters signify the respective classes.

Both qualitative and quantitative analyses demonstrate ADEL’s superior discriminative power. t-SNE visualizations (Figure 4) show well-separated clusters with exceptional intra-class compactness and inter-class separation. This is quantitatively validated by SC analysis, where ADEL achieves the highest 0.9184 SC. The performance hierarchy reveals traditional methods (CNNEL: 0.8558; multi-feature fusion: 0.8734), hybrid approach (CVCNN: 0.9021), advanced architectures (GLFormer: 0.8701, CNN/VGG: 0.9145) and our ADEL framework (0.9184). Notably, ADEL’s 0.0039 SC advantage over CNN/VGG suggests fundamental architectural improvements rather than incremental gains.

To further validate ADEL’s effectiveness, we conducted comprehensive experiments using a LoRa dataset. The acquisition of LoRa employed a USRP N210 software-defined radio (SDR) platform as the receiver, operating at a carrier frequency of 868.1 MHz with a transmission interval of 0.3 s. The receiver sampling rate was configured at 1 MHz. For this evaluation, we utilized a representative subset comprising 20 device classes, with 500 samples per class. The dataset was partitioned using random sampling into training (60%), validation (20%) and testing (20%) subsets to ensure rigorous performance assessment. The full experimental dataset details are available in [56].

**Figure 5:**
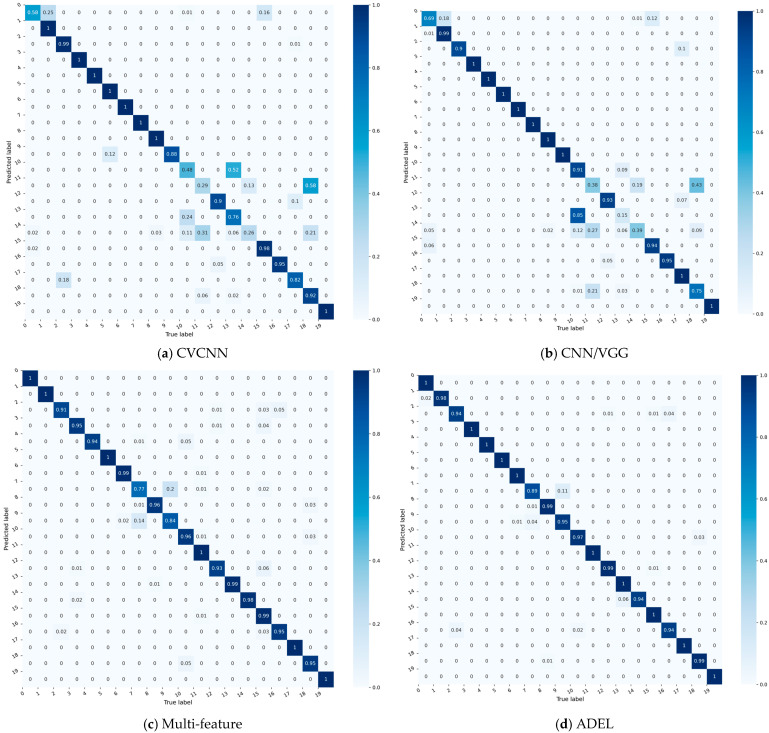
The comparative results of confusion matrices on LoRa dataset.

Among the aforementioned models achieving accuracy exceeding 80%, CVCNN, CNN/VGG, multi-feature and ADEL attain accuracies of 84.05%, 84.90%, 95.50% and 97.90%, respectively, on the LoRa dataset. Their corresponding confusion matrices are presented in Figure 5. Although these accuracy values are lower than those achieved on the ADS–B dataset, ADEL still outperforms the multi-feature approach by 2.40%.

**Figure 6:**
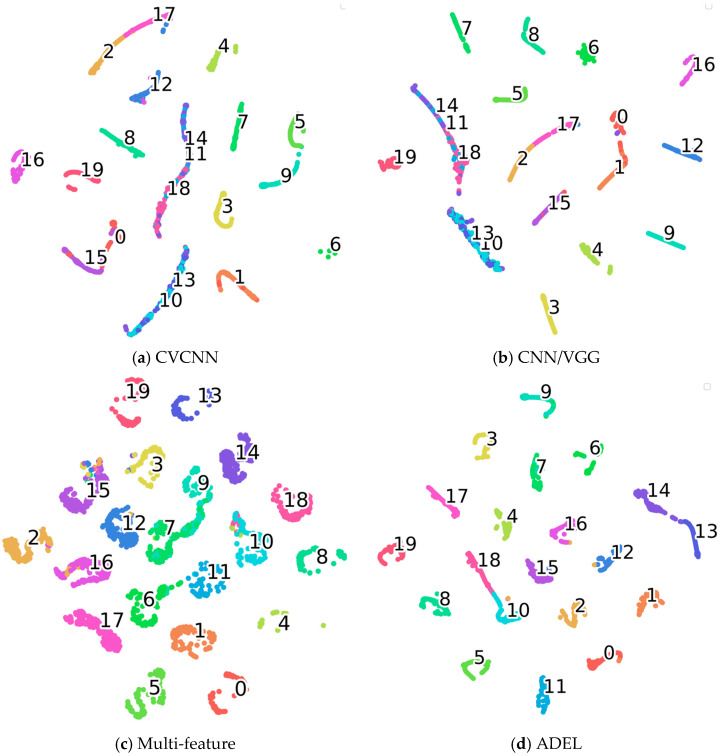
The t-SNE results of learned features on LoRa dataset.

Figure 6 presents t-SNE visualizations of the learned feature representations for each network architecture. The corresponding silhouette coefficients (SC) are 0.6235, 0.6289, 0.9035 and 0.9123, respectively. Higher SC values indicate superior feature representation quality, which is corroborated by the tighter cluster separation and reduced inter-class overlap observed in the t-SNE projections.

Table 8 presents the parameter counts and inference latency metrics across the evaluated models. The results demonstrate that while ADEL achieves superior performance relative to comparative approaches, this performance advantage comes at the cost of substantially increased model complexity and longer inference times. These computational considerations warrant careful attention in resource-constrained deployment scenarios.

### 3.3. Ablation Results

The ADEL framework incorporates the following three core optimization components: categorical cross-entropy loss, center loss and mean squared error (MSE). When MSE is employed, it concurrently activates adaptive weighted inference for ensemble predictions. Ablation study results quantifying these components’ contributions are presented in Table 9, where the symbol ✓ denotes component inclusion and ✗ indicates exclusion.

Table 9 reveals substantial performance improvements through hybrid loss optimization. Baseline cross-entropy loss alone yields 93.00% accuracy, while integrating MSE loss (λ = 0.49) elevates accuracy by 4.55% to 97.55%, demonstrating the critical role of signal reconstruction fidelity. Augmenting center loss (λ_center_ = 0.56) achieves comparable enhancement (97.10% accuracy), highlighting its efficacy in compressing intra-class variance. Crucially, simultaneously incorporating both auxiliary losses (λ = 0.31, λ_center_ = 0.85) establishes peak accuracy at 99.75%, a 6.75% improvement over baseline—validating their complementary mechanisms in refining feature space geometry.

**Figure 7:**
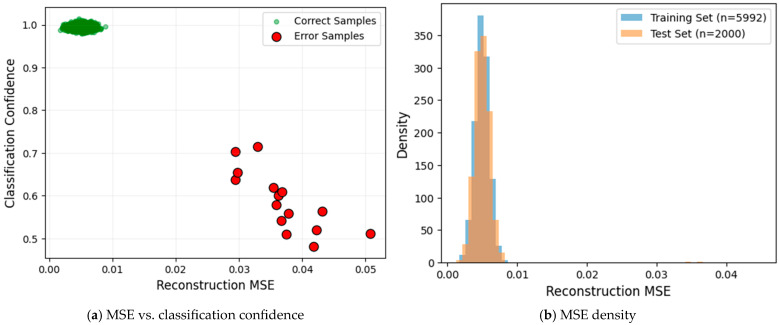
The validation of MSE’s role in ADEL.

The adaptive weighting mechanism based on reconstruction error (MSE) plays a pivotal role in ADEL’s performance. As illustrated in Figure 7a, we observe a strong negative correlation between MSE and classification confidence as follows: correctly classified samples predominantly cluster in the low-MSE/high-confidence region, while few misclassified samples exhibit significantly higher MSE values. This distinct separation confirms that reconstruction quality reliably indicates classification reliability. Furthermore, Figure 7b demonstrates statistically equivalent MSE distributions between training and test sets. This distributional alignment provides robust evidence that the model learns transferable features rather than memorizing training set artifacts.

**Figure 8:**
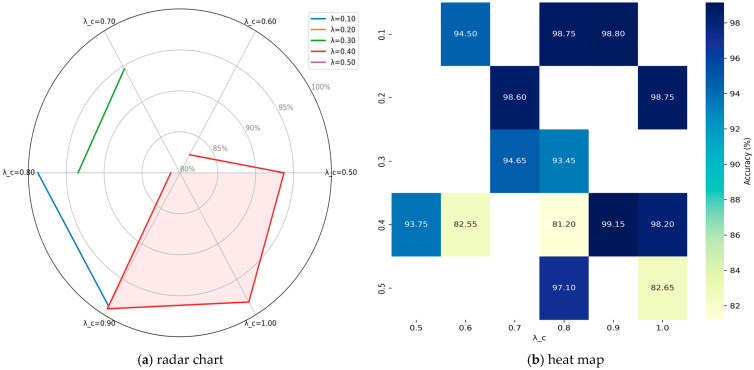
The parameter sensitivity analysis of ADEL.

To quantitatively assess the individual contributions of each component in our hybrid loss function (Equation (1)), we perform a comprehensive parameter sensitivity analysis. As demonstrated in Figure 8, the model’s classification accuracy (accuracy) is systematically evaluated across $\lambda \in \left[0.1,0.5\right]\left(\Delta \lambda =0.1\right)$ and $\lambda_{center}\in \left[0.5,1\right]\left(\Delta \lambda_{center}=0.1\right)$, with all configurations achieving > 80% accuracy being visualized through both radar chart and heatmap representations.

The radar chart and heatmap analysis reveals a systematic evaluation of parameter interactions between λ and λ_c. The visualization demonstrates that model performance exhibits strong dependence on both parameters, with particularly notable behavior observed for the λ = 0.40 configuration (red polygon), which achieves optimal accuracy (99.15%) at λ_c = 0.90; this represents a significant 17.95% improvement over the λ = 0.40 configuration (81.20% at λ_c = 0.80). The heatmap quantitatively validates these findings, showing a well-defined high-performance region (dark blue cells) concentrated at λ = 0.10–0.20 with λ_c ≥ 0.70. Three distinct performance tiers emerge: (1) a peak performance zone (accuracy > 99%) centered at (λ = 0.40, λ_c = 0.90); (2) a transition zone (94–97%) surrounding the peak region. Notably, the λ = 0.40 configuration exhibits anomalous behavior, with accuracy dropping sharply to 81.20% at λ_c = 0.80 despite reasonable performance at higher λ_c values, suggesting complex parameter interactions. The concordance between these complementary visualization techniques strongly supports the conclusion that the λ = 0.40/λ_c = 0.90 combination represents the optimal parameter configuration, while also highlighting the importance of avoiding the λ = 0.40 regime when λ_c approaches 0.80. Notably, when implementing a more granular search interval (0.01 versus 0.10), the model maintains its peak performance capability, consistently reaching 99.75% accuracy under optimal parameter combinations.

**Figure 9:**
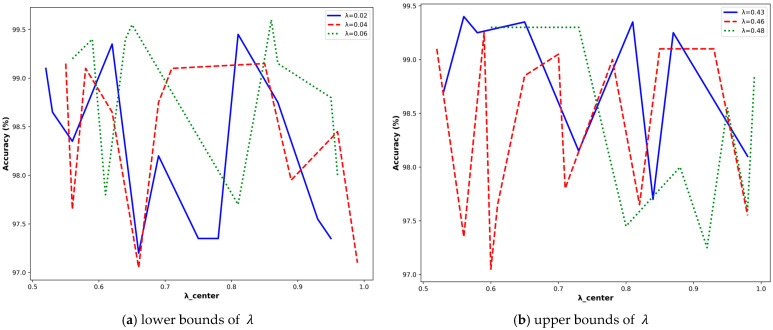
The fine-grained results of λ and λ_center.

To better characterize the behavior of these parameters, we performed a fine-grained grid search over λ ∈ [0.1,0.5] (∆λ = 00.1) and λ_center ∈ [0.5,1] (∆λ_center = 0.01). As shown in Figure 9, despite persistent fluctuations in performance, classification accuracy consistently exceeds 97% across both lower and upper bounds of λ values.

## 4. Discussion

The proposed ADEL framework represents a significant advancement in specific emitter identification (SEI) through its innovative integration of the following three key components: (1) heterogeneous deep network architectures, (2) hybrid loss optimization, and (3) adaptive ensemble inference based on signal reconstruction error. Our experimental results demonstrate improvements over existing methods with an average accuracy enhancement of 2.55% and 4% compared to state-of-the-art approaches [48,53] respectively.

These findings align with but substantially extend previous work in two important dimensions. First, while prior studies have explored ensemble methods for SEI, ADEL’s novel reconstruction-error-based adaptive weighting mechanism addresses the critical limitation of static ensemble approaches identified in [46,48]. Second, our hybrid loss function, which combines margin-based and reconstruction-based terms, provides a mathematical framework that unifies and generalizes the separate loss strategies proposed in [57,58].

The implications of these results extend beyond SEI applications. The adaptive ensemble strategy may be particularly valuable for other time series classification tasks where input quality varies significantly, such as biomedical signal processing [59,60] or industrial equipment monitoring [61,62]. Furthermore, the success of our hybrid loss formulation suggests promising directions for developing unified loss functions that simultaneously optimize for discriminative and reconstructive objectives.

Future research should investigate the generalizability of ADEL’s architecture to other radio frequency fingerprinting scenarios and hardware-efficient implementations for edge-device deployment.

## Figures and Tables

**Table 1:** The structure of the designed CNN base learner.

Layer (Activation)	Hyperparameters	Value	Detailed Descriptions
Conv1(ReLU)	*N_F_*_1_	2/48	The input and output channels
	*N_K_*_1_	5	The kernel size
MaxPooling1	*N_D_*_1_	2	The down-sampling factor
	*N_S_*_1_	2	The pooling stride
Conv2(ReLU)	*N_F_*_2_	48/48	The input and output channels
	*N_K_*_2_	5	The kernel size
MaxPooling2	*N_D_*_2_	2	The down-sampling factor
	*N_S_*_2_	2	The pooling stride
Conv3(ReLU)	*N_F_*_3_	48/48	The input and output channels
	*N_K_*_3_	5	The kernel size
MaxPooling3	*N_D_*_3_	2	The down-sampling factor
	*N_S_*_3_	2	The pooling stride
Conv4(ReLU)	*N_F_*_4_	48/48	The input and output channels
	*N_K_*_4_	5	The kernel size
MaxPooling4	*N_D_*_4_	2	The down-sampling factor
	*N_S_*_4_	2	The pooling stride

**Table 2:** The structure of the designed MLP base learner.

Layer (Activation)	Hyperparameters	Value	Detailed Descriptions
Conv1(ReLU)	*N_F_*_1_	2/48	The input and output channels
	*N_K_*_1_	5	The kernel size
MaxPooling1	*N_D_*_1_	2	The down-sampling factor
	*N_S_*_1_	2	The pooling stride
Linear1(ReLU)	*N_H_*_1_	750	Units of hidden layer
Linear2	*N_H_*_2_	375	Units of hidden layer

**Table 3:** The structure of the designed transformer base learner.

Layer (Activation)	Hyperparameters	Value	Detailed Descriptions
Conv1(ReLU)	*N_F_*_1_	2/48	The input and output channels
	*N_K_*_1_	5	The kernel size
MaxPooling1	*N_D_*_1_	2	The down-sampling factor
	*N_S_*_1_	2	The pooling stride
Transformer encoder	*N_d_model_*	64	Model dimensionality
	*N_nhead_*	8	Number of attention heads
	*N_layers_*	1	Number of transformer blocks
	*N_dim_forward_*	384	Feedforward network dimension

**Table 4:** The structure of the shared classifier.

Layer (Activation)	Hyperparameters	Value	Detailed Descriptions
Linear1(ReLU)	*N_H_*_1_	512	Units of hidden layer
Linear2	*N_H_*_2_	20	Number of classes

**Table 5:** The structure of the shared decoder.

Layer (Activation)	Hyperparameters	Value	Detailed Descriptions
Linear1	*N*_*H*1_	750	Units of hidden layer
ConvTranspose1	*N_F_*_1_	48/48	The input and output channels
	*N*_*K*1_	3	The kernel size
BatchNorm1	*N*_*B*1_	48	The input channels
ReLU1	✗	✗	Activation function
UpSample1	*N*_*s*1_	2	Up-sampling factor
ConvTranspose2	*N_F_*_2_	48/24	The input and output channels
	*N*_*K*2_	3	The kernel size
BatchNorm2	*N*_*B*2_	24	The input channels
ReLU2	✗	✗	Activation function
UpSample2	*N*_*s*2_	2	Up-sampling factor
ConvTranspose3	*N_F_*_3_	24/2	The input and output channels
	*N*_*K*3_	3	The kernel size
BatchNorm3	*N*_*B*3_	2	The input channels
UpSample3	*N*_*s*3_	2	Up-sampling factor

**Table 6:** The benchmarking results in terms of the evaluation metrics on ADS–B dataset.

Methods	Accuracy (%)	Precision (%)	Recall (%)	Macro-F1 (%)
MCNet [50]	87.12 ± 1.42	89.53 ± 1.31	87.12 ± 1.42	86.68 ± 1.52
PETCGDNN [51]	80.76 ± 6.32	83.59 ± 6.02	80.76 ± 6.32	78.94 ± 6.30
CNN/VGG [52]	96.58 ± 0.89	96.78 ± 0.90	96.58 ± 0.89	96.56 ± 0.92
MSCANet [53]	86.94 ± 1.29	89.92 ± 0.76	86.94 ± 1.29	85.21 ± 1.25
CVCNN [40]	93.93 ± 1.08	94.75 ± 1.05	93.93 ± 1.08	92.74 ± 1.37
FFTMHA [54]	72.82 ± 1.45	74.67 ± 1.51	72.82 ± 1.49	71.78 ± 1.35
CEL [45]	77.98 ± 1.34	79.02 ± 1.14	77.98 ± 1.34	77.85 ± 1.26
CNNEL [46]	93.95 ± 1.12	95.52 ± 0.72	93.95 ± 1.12	92.98 ± 1.55
HMSFF [47]	81.35 ± 0.58	84.62 ± 0.73	81.35 ± 0.58	79.75 ± 0.61
Multi-feature [48]	93.08 ± 2.24	93.89 ± 2.08	93.08 ± 2.24	92.51 ± 2.72
GLFormer [32]	93.70 ± 1.09	94.59 ± 0.79	93.70 ± 1.09	92.98 ± 1.0
ADEL	98.25 ± 0.69	98.39 ± 0.61	98.25 ± 0.68	98.26 ± 0.69

**Table 7:** The comparative results of silhouette scores.

Methods	CNNEL	Multi-Feature	CVCNN	CNN/VGG	GLFormer	ADEL
**SC**	0.8558	0.8734	0.9021	0.9145	0.8701	0.9184

**Table 8:** The comparative results of silhouette scores.

Methods	Multi-Feature	CVCNN	CNN/VGG	ADEL
**Parameters (K)**	1154	1402	569	3064
**Inference latency (s)**	1.18	0.59	0.42	0.95

**Table 9:** The ablation results of ADEL.

Components	Cross-Entropy Loss	Center Loss	MSE	Accuracy
1	✓	✗	✗	93.00%
2	✓	✗	✓	97.55%
3	✓	✓	✗	97.10%
4	✓	✓	✓	99.75%

## Data Availability

The data presented in this study can be obtained via the website https://github.com/BeechburgPieStar/FS-SEI, accessed on 1 October 2025.

## Abbreviations

The following abbreviations are used in this manuscript:

MCNet	Modulation classification network
DNN	Deep neural network
PETCGDNN	Parameter estimation and transformation-based CNN-gated recurrent unit DNN
VGG	Visual geometry group
MSCANet	Multi-scale convolutional attention network
FFTMHA	Fast Fourier transform with multihead attention
RNN	Recurrent neural network
